# Dependability of Entrustable Professional Activity Portfolios in Otorhinolaryngology Residency: Nationwide Generalizability and Decision Study

**DOI:** 10.2196/104628

**Published:** 2026-10-05

**Authors:** Jeng-Wen Chen, Ching-Lin Hsieh, Chun-Hsiang Chang, Ya-Hui Wang, Wei-Chung Hsu, Pa-Chun Wang, Li-Jen Liao, Chun-Hou Liao, Mingchih Chen, Okki Dhona Laksmita

**Affiliations:** 1 Department of Otorhinolaryngology–Head and Neck Surgery, Cardinal Tien Hospital and Fu Jen Catholic University, 362, ZhongZheng Rd. Xindian Dist., New Taipei City, 23148, Taiwan, 886 2-22193391 ext 67451; 2 Department of Hospital Management, Graduate Institute of Business Administration, Fu Jen Catholic University, New Taipei City, Taiwan; 3 Department of Otorhinolaryngology–Head and Neck Surgery, National Taiwan University Hospital, Taipei, Taiwan; 4 School of Occupational Therapy, College of Medicine, National Taiwan University, Taipei, Taiwan; 5 Department of Physical Medicine and Rehabilitation, National Taiwan University Hospital, Taipei, Taiwan; 6 Department of Occupational Therapy, College of Medical and Health Sciences, Asia University, Taichung, Taiwan; 7 Medical Research Center, Cardinal Tien Hospital and Fu Jen Catholic University, New Taipei City, Taiwan; 8 Sleep Center, National Taiwan University Hospital, Taipei, Taiwan; 9 Department of Otolaryngology, College of Medicine, National Taiwan University, Taipei, Taiwan; 10 Department of Otolaryngology, Cathay General Hospital, Taipei, Taiwan; 11 Department of Otolaryngology, School of Medicine, Fu-Jen Catholic University, New Taipei City, Taiwan; 12 Department of Medical Research, China Medical University Hospital, Taichung, Taiwan; 13 Department of Otolaryngology, Far Eastern Memorial Hospital, New Taipei City, Taiwan; 14 Division of Urology, Department of Surgery, Cardinal Tien Hospital and Fu Jen Catholic University, New Taipei City, Taiwan; 15 Artificial Intelligence Development Center, Fu Jen Catholic University, New Taipei City, Taiwan

**Keywords:** entrustable professional activities, competency-based medical education, generalizability theory, clinical competency committee, postgraduate medical education

## Abstract

**Background:**

Competency-based medical education increasingly uses digital workplace assessment platforms to collect longitudinal evidence of trainees’ readiness for progressive responsibility. Entrustable professional activities (EPAs) translate competencies into observable clinical work, but individual workplace observations are context-dependent and may not support dependable progression decisions. Empirical guidance remains limited on how many observations, faculty raters, EPA titles, and workplace settings are needed for a dependable EPA portfolio.

**Objective:**

This study aimed to estimate EPA portfolio dependability for competency-based progression review using nationwide data from a digital workplace assessment platform in otorhinolaryngology-head and neck surgery residency training.

**Methods:**

We conducted a retrospective, nationwide cross-sectional generalizability and decision study using routinely collected EPA-based workplace assessment data from Taiwan’s E-MyWay platform. The study included completed EPA observations from accredited otorhinolaryngology-head and neck surgery residency programs between August 2022 and May 2026. Faculty-assigned entrustment-supervision ratings were scored on a 5-level national EPA scale. The resident was the object of measurement, whereas faculty rater, EPA title, and workplace setting were measurement facets. The generalizability study partitioned rating variance into resident, facet, interaction, and residual components, which were subsequently used in decision-study projections of the generalizability coefficient, Phi coefficient, and absolute standard error of measurement under alternative portfolio configurations.

**Results:**

The analytic sample included 45,526 EPA observations involving 466 residents and 448 faculty raters across 35 training programs, 11 EPA titles, and 5 workplace settings. Residents had a median of 87 (IQR 47-124) observations and were assessed by a median of 9 (IQR 6-12) faculty raters across 11 (IQR 10-11) EPA titles and 5 (IQR 4-5) workplace settings. Single EPA observations were insufficient for resident-level summative interpretation. In the primary generalizability study, residual/encounter-level variance was the largest component, accounting for 40.1% of total variance; resident variance accounted for 26.9%, faculty rater variance accounted for 10.8%, and resident × faculty rater interaction for 10.4%. A study-defined well-distributed 10-observation portfolio achieved a generalizability coefficient of 0.77 but an absolute dependability coefficient (Phi) of 0.65. A 20-observation portfolio across 5 faculty raters, 5 EPA titles, and 3 workplace settings yielded a Phi of 0.76, and a 30-observation portfolio across 6 faculty raters, 6 EPA titles, and 4 workplace settings yielded a Phi of 0.80.

**Conclusions:**

Nationwide digital workplace assessment data showed that EPA portfolio dependability reflected both observation counts and sampling breadth across faculty raters, EPA titles, and workplace settings. These study-defined portfolio configurations may help clinical competency committees, residency programs, and specialty societies monitor portfolio completeness before progression deliberation. They should complement, rather than replace, narrative feedback, case complexity, performance trajectory, and committee judgment.

## Introduction

Digital workplace assessment platforms are increasingly used to collect longitudinal evidence for progression decisions [1-3]. In postgraduate competency-based medical education (CBME) [4,5], these systems can capture entrustable professional activity (EPA) [6,7] observations across faculty raters, clinical tasks, and workplace settings [8]. However, the availability of large volumes of digital assessment data does not by itself ensure that resident portfolios contain sufficient, well-distributed evidence for defensible progression review [9,10].

A persistent challenge for digital EPA systems is determining when accumulated workplace observations become an adequate evidence portfolio [11,12]. Individual EPA observations are useful for formative feedback, but summative progression decisions require aggregation across contextually variable encounters [13]. Without empirical benchmarks, programs may under-sample performance, risking unstable decisions, or over-sample, increasing assessment burden without proportional gains in decision quality [14].

Digital platforms create an opportunity to examine these sampling problems at scale by preserving structured information about raters, EPA titles, workplace settings, and longitudinal assessment exposure. Otorhinolaryngology-head and neck surgery is an informative specialty context, with residents assessed across emergency, inpatient, outpatient, procedural, and operating-room environments [15,16]. This breadth makes the specialty well suited for studying how EPA portfolios should be sampled across clinical tasks and settings. Taiwan offers a unique national context because, since 2022, the Taiwan Society of Otorhinolaryngology-Head and Neck Surgery (TSO-HNS) has implemented a specialty EPA framework through the E-MyWay platform across accredited residency programs, enabling standardized national capture of EPA ratings, faculty identifiers, resident identifiers, EPA titles, and workplace settings [17-20]. This infrastructure allows portfolio dependability to be examined as a learning analytics question rather than as a single-program quality assurance exercise [21,22].

This study used nationwide E-MyWay data [17-20] to examine EPA portfolio dependability as a multidimensional sampling problem in CBME [4-7,14]. Rather than focusing only on the number of EPA observations, we evaluated how resident-level portfolio dependability varied jointly with observation count and breadth across faculty raters, EPA titles, and workplace settings [23,24]. By combining routine digital workplace-based assessment data with generalizability study (G-study) and decision study (D-study) modeling [23-25], this study aimed to provide portfolio-design evidence for programmatic assessment in competency-based otorhinolaryngology residency training [2,9,26].

## Methods

### Study Design and Participants

We conducted a retrospective, nationwide cross-sectional G-study and D-study using routinely collected EPA-based workplace assessment data from Taiwan’s E-MyWay platform [20]. The study evaluated whether faculty-assigned entrustment-supervision ratings, aggregated across resident assessment portfolios, provided dependable evidence for resident-level progression review in otorhinolaryngology-head and neck surgery residency training. The intended interpretation was resident entrustment readiness across the specialty EPA framework; the intended use was programmatic review and progression decision-making.

Otorhinolaryngology-head and neck surgery residency training in Taiwan lasts 4.5 years, spanning postgraduate year 3 through postgraduate year 7. During the study period, all 35 accredited programs using E-MyWay were included, representing the national implementation network for this specialty. Participating programs were distributed across all 6 National Health Insurance Administration regional divisions: Taipei (n=16), Northern (n=2), Central (n=7), Southern (n=4), Kao-Ping (n=5), and Eastern (n=1), reflecting broad national coverage of accredited training sites.

### Digital Workplace Assessment Platform

E-MyWay is a web app–based national CBME platform, developed by the Joint Commission of Taiwan, to capture structured EPA assessment records [20]. In otorhinolaryngology-head and neck surgery, it enables standardized documentation of resident identifier, faculty rater identifier, EPA title, workplace setting, entrustment-supervision rating, narrative resident reflection, and faculty feedback [18]. The TSO-HNS developed the framework through an iterative national expert-consensus process beginning in August 2020. A CBME workgroup comprising residency review committee members, program directors, specialty experts, and physician educators used Glaser’s modified state-of-the-art approach, followed by internal and external quantitative and qualitative review. The framework was finalized in June 2021, and stage-specific expected entrustment-supervision levels were agreed upon during pilot implementation in 2021 to 2022. This process was an expert-consensus and pilot-refinement process rather than a formal Delphi study. The platform supports program-level monitoring and permits aggregation of EPA observations into resident portfolios; the expectations are summarized in Table 1 [20]. The expected levels were provided for curricular context only and were not used to transform observed ratings or calculate the G-study or D-study estimates.

**Table 1.** Distribution of entrustable professional activity (EPA)–based workplace assessment records across 11 EPA titles and expected entrustment-supervision levels by resident seniority.

EPA^a^ title	Assessment records, n (%)^b^	A priori expected entrustment-supervision levels by resident seniority^c^				
		PGY3^d^	PGY4	PGY5	PGY6	PGY7
EPA01 (Airway) Assessing and managing patients with airway presentations	3693 (8.1)	2	3	3	4	5
EPA02 (Foreign Body) Assessing and managing patients with suspicious foreign body presentations	3263 (7.2)	2	3	3	4	5
EPA03 (Bleeding) Assessing and managing patients with upper aerodigestive tract bleeding presentations	3139 (6.9)	2	3	3	4	5
EPA04 (Vertigo) Assessing and managing patients with vertigo	2346 (5.2)	2	3	3	4	5
EPA05 (Infection) Assessing and managing patients with head and neck infections	4033 (8.9)	2	3	3	4	5
EPA06^e^ (Head and Neck) Assessing and managing patients with head and neck masses	7935 (17.4)	2	2	3	4	5
EPA07 (Ear) Assessing and managing patients with ear and hearing diseases	5521 (12.1)	2	3	3	4	5
EPA08 (Sinonasal) Assessing and managing patients with sinonasal diseases	6071 (13.3)	2	3	4	4	5
EPA09 (Larynx) Assessing and managing patients with laryngopharyngeal diseases (voice/speech/language/dysphagia)	3729 (8.2)	2	3	3	4	5
EPA10^e^ (Sleep Disordered Breathing) Assessing and managing patients with sleep-disordered breathing	3443 (7.6)	2	2	3	4	5
EPA11^e^ (Plasty) Assessing and managing patients with facial plastic and reconstructive surgery	2353 (5.2)	2	2	3	3	4

a EPA: entrustable professional activity.

b The analytic dataset included EPA01-EPA11 only. EPA12 was present in the original data source but was excluded owing to its later introduction and inconsistent availability across the analytic window.

c Expected entrustment-supervision levels were established a priori through the Taiwan Society of Otorhinolaryngology-Head and Neck Surgery iterative expert-consensus and pilot-refinement process. They represent curricular reference expectations for each integrated EPA by training stage, rather than phase-specific or empirically derived cut points.

d PGY: postgraduate year.

e Delayed EPAs: EPA06, EPA10, and EPA11.

### Portfolio Definitions

An observation was defined as a completed EPA-based workplace assessment record. A resident assessment portfolio was defined as the accumulated set of EPA observations for an individual resident. Portfolio size referred to the number of observations, whereas portfolio breadth referred to sampling across faculty raters, EPA titles, and workplace settings. The dataset had an unbalanced, partially crossed, multi-institutional structure, with residents and faculty raters largely clustered within training hospitals but partially crossed through workplace assessments.

### Data Source

We analyzed EPA-based workplace assessment records from the August 2022 to May 2026 academic years. The data extraction date was May 1, 2026. Each record represented one completed workplace observation in which a faculty rater assessed the component of an EPA demonstrated during a specific clinical encounter and workplace setting and assigned an entrustment-supervision level for that observed performance.

The primary analysis included EPA01 through EPA11, which constituted the consistently implemented specialty EPA framework during the study period [17]. EPA12 was excluded owing to its introduction in 2024 and inconsistent availability across the analytic window. Conference-room assessments were also excluded because they represented case presentations rather than true workplace-based assessments. The eligible workplace settings were operating room, ward, including intensive care unit, emergency department, outpatient clinic, and consultation. Records were included if they contained complete data for entrustment–supervision level, resident identifier, faculty rater identifier, EPA title, and workplace setting.

### Outcome and Measurement Facets

The primary outcome was the faculty-assigned entrustment-supervision rating, scored on a 5-level scale aligned with the national specialty EPA framework [6,20]. The 5-level scale used common supervision anchors: observation without active participation, direct and proactive supervision, indirect and reactive supervision, independent practice, and supervision of junior learners. Higher scores indicated greater readiness for less supervised practice. The resident was specified as the object of measurement to align with the intended inference of resident-level EPA portfolio dependability for programmatic review and progression deliberation. Faculty rater, EPA title, and workplace setting were treated as measurement facets given their roles as major sources of sampling variation in routine workplace assessment. EPA title was included as a facet to examine aggregate portfolio dependability across the specialty EPA framework rather than certification-level dependability for each individual EPA.

Selected 2-way interactions—resident × faculty rater, resident × EPA title, and faculty rater × EPA title—were modeled to capture major sources of score variation. Residual/encounter-level variance was interpreted as remaining assessment-level or encounter-level variation not explained by the modeled resident, faculty rater, EPA title, workplace setting, or interaction components. This component may include occasion-specific resident performance, patient or case factors, unmodeled contextual variation, higher-order interactions, rater inconsistency, and measurement error. The same clinical encounter was not independently rated by multiple faculty raters; therefore, residual/encounter-level variance was not interpreted as conventional interrater reliability.

Although entrustment-supervision ratings are ordinal, treating them as approximately numeric is therefore a modeling assumption. We used this approach to support variance decomposition and D-study projection, and we interpreted the results as estimates of portfolio dependability rather than direct measures of clinical competence.

### G-Study

We conducted a G-study to decompose variance in entrustment-supervision ratings [25]. Consistent with the measurement-facet framework described above, the primary model was an intercept-only random-effects linear mixed-effects model that included random components for resident, faculty rater, EPA title, workplace setting, resident × faculty rater, resident × EPA title, faculty rater × EPA title, and residual error. This model was selected to estimate aggregate resident-level portfolio dependability across the specialty EPA framework rather than EPA-specific dependability for each individual EPA. The full model specification is provided in Multimedia Appendix 1. Given the unbalanced, partially crossed, multi-institutional data structure, variance components were interpreted in relation to the intended resident-level inference rather than as properties of individual observation records. Variance components were expressed as proportions of total variance to quantify resident signal, rater effects, task effects, workplace effects, interaction effects, and encounter-level variation.

### D-Study

Using variance components from the primary G-study, we conducted D-studies to estimate the projected dependability of alternative EPA portfolio configurations [23,24,27,28]. The Phi coefficient was emphasized as the primary dependability index for absolute decisions, consistent with the criterion-referenced nature of resident-level programmatic review and progression deliberation. The generalizability coefficient was reported secondarily as an index of relative dependability, and the absolute standard error of measurement (SEM) was calculated to quantify expected measurement error in the original rating metric.

D-study scenarios varied the number of observation records, faculty raters, EPA titles, and workplace settings to model both portfolio size and breadth. The D-study portfolio configurations were specified as practical, study-defined scenarios in which portfolio size and breadth increased together. The 10-, 20-, 30-, 40-, 60-, median observed, and 100-observation scenarios were selected to represent a range of plausible portfolio structures, from smaller portfolios for routine programmatic review to broader portfolios that may be considered before more consequential progression deliberation. These configurations were not empirically optimized cutoffs or validated progression standards; rather, they were model-based scenarios used to examine how projected dependability changed when observations were distributed across increasing numbers of faculty raters, EPA titles, and workplace settings. Phi ≥0.70 and Phi ≥0.80 were used as prespecified psychometric reference points for interpreting projected dependability, not as validated standards for competence, certification, or progression.

### Validity Framework

We interpreted dependability as one source of validity evidence for using EPA scores in progression review [26]. Within the Messick framework [29], this study focused on internal structure evidence by examining how much variation in entrustment ratings was attributable to residents relative to measurement facets and residual error. We did not evaluate other sources of validity evidence, including content, response processes, relations to other variables, or consequences [30]. Accordingly, the findings should be interpreted as one component of a broader validity argument for EPA-based progression decisions [26,31-34].

### Portfolio Concentration

To characterize portfolio imbalance arising from opportunistic workplace-based assessment, we conducted a descriptive portfolio concentration analysis. For each resident, we calculated the proportion of observations contributed by the most frequent faculty rater, the most frequent EPA title, and the most frequent workplace setting. These resident-level proportions were summarized using medians and interquartile ranges. This analysis was intended to describe, but not statistically correct, observed sampling imbalance.

### Sensitivity Analyses

We performed 3 sensitivity analyses to examine model robustness. First, an EPA title × workplace setting sensitivity model assessed whether task-context combinations materially changed the variance-component structure. Second, to address potential hospital or program-level effects, we fitted a hospital-aware sensitivity model that added random intercepts for resident hospital and faculty hospital to the primary G-study model. Hospital codes were modeled separately for residents and faculty raters to reflect the separate recording of resident and faculty hospital affiliations in the dataset. This model was used to evaluate whether between-hospital differences, including potential differences in curriculum, clinical exposure, assessment culture, or rater stringency, materially changed the variance-component structure. Hospital was treated as a contextual sensitivity specification rather than a primary measurement facet, consistent with the intended inference of aggregate resident-level portfolio dependability across the specialty EPA framework rather than hospital comparison.

Third, to examine whether case complexity accounted for residual/encounter-level variation, we fitted a complexity-adjusted sensitivity model that added case complexity as a fixed effect to the primary G-study model. Case complexity was available for 45,513 of 45,526 observations; 31,148 (68.4%) observations were classified as basic/routine and 14,365 (31.6%) as advanced/nonroutine, with 13 observations missing case-complexity data. The 13 observations with missing case-complexity data were excluded from this sensitivity analysis, yielding an analytic sample of 45,513 observations. This model was used to assess whether adjustment for case complexity materially changed the variance-component structure.

### Statistical Analysis

All analyses were conducted using R (version 4.5.3; R Foundation for Statistical Computing). Data management and descriptive analyses used the tidyverse package, and linear mixed-effects models were estimated with lme4. D-study projections were derived from the estimated variance components. Observed resident portfolios were summarized by comparing each resident’s assessment portfolio with the study-defined portfolio configurations for Phi ≥0.70 and Phi ≥0.80.

### Ethical Considerations

This study was approved by the Institutional Review Board of Cardinal Tien Hospital (CTH-114-3-5-019). All data were analyzed in deidentified form. Electronic consent for research use was obtained at initial E-MyWay platform enrollment and activation. Data were obtained from the TSO-HNS E-MyWay database with permission. The study is reported in accordance with applicable STROBE (Strengthening the Reporting of Observational Studies in Epidemiology) guidance for observational studies, with adaptations for G-study and D-study reporting (Checklist 1).

## Results

### Resident Assessment Exposure

The E-MyWay platform yielded 45,526 complete EPA-based workplace assessment records from 466 residents and 448 faculty raters across 35 accredited training programs, covering 11 EPA titles and 5 workplace settings. Residents had a mean of 97.7 (SD 64.9) observations and a median of 87 (IQR 47‐124; range, 1‐501) observations. Each resident was assessed by a median of 9 (IQR 6‐12) faculty raters, across a median of 11 (IQR 10‐11) EPA titles, and in a median of 5 (IQR 4‐5) workplace settings.

EPA-specific assessment volume is summarized in Table 1. The most frequently assessed EPAs were EPA06, head and neck/oral mass assessment and management (n=7935 observations, 17.4%); EPA08, sinonasal disease assessment and management (n=6071, 13.3%); and EPA07, ear and hearing disease assessment and management (n=5521, 12.1%). The least frequently assessed were EPA04, vertigo assessment and management (n=2346, 5.2%), and EPA11, facial plastic and reconstructive surgery assessment and management (n=2353, 5.2%).

### Variance Components From the G-Study

Variance components from the primary G-study are presented in Table 2. Residual/encounter-level variance was the largest component, accounting for 40.1% of total variance. Resident variance accounted for 26.9%, faculty rater variance accounted for 10.8%, and the resident × faculty rater interaction accounted for 10.4%. EPA title, workplace setting, resident × EPA title interaction, and faculty rater × EPA title interaction accounted for 4.2%, 2.5%, 2.0%, and 3.1%, respectively.

**Table 2.** Variance components from the generalizability study model.

Variance component^a^	Variance	Total variance (%)
Resident	0.32	26.9
Faculty rater	0.13	10.8
EPA^b^ title	0.05	4.2
Workplace setting	0.03	2.5
Resident × faculty rater	0.12	10.4
Resident × EPA title	0.02	2.0
Faculty rater × EPA title	0.04	3.1
Residual/encounter-level variance	0.47	40.1

a Resident variance represents universe-score variance. Resident × faculty rater, resident × EPA title, and residual variance contribute to relative error variance. Absolute error variance additionally includes faculty rater, EPA title, workplace setting, and faculty rater × EPA title variance after adjustment for the corresponding numbers of raters, EPA titles, workplace settings, and observations in each projected portfolio.

b EPA: entrustable professional activity.

### D-Study Portfolio Projections

D-study projections are summarized in Table 3. A well-distributed 10-observation portfolio across 3 faculty raters, 3 EPA titles, and 2 workplace settings yielded a G coefficient of 0.77 but a Phi coefficient of 0.65 and an absolute SEM of 0.42.

A study-defined well-distributed 20-observation portfolio across 5 faculty raters, 5 EPA titles, and 3 workplace settings yielded a G coefficient of 0.86, a Phi coefficient of 0.76, and an absolute SEM of 0.32, reaching the prespecified Phi ≥0.70 psychometric reference point. A study-defined well-distributed 30-observation portfolio across 6 faculty raters, 6 EPA titles, and 4 workplace settings yielded a G coefficient of 0.89, a Phi coefficient of 0.80, and an absolute SEM of 0.28, reaching the stricter Phi ≥0.80 psychometric reference point. Thus, increasing the number of observations without adequate distribution across raters, EPAs, and settings was insufficient to ensure higher absolute dependability. Together, these model-based projections showed higher absolute dependability in portfolio configurations with larger observation counts and broader sampling across faculty raters, EPA titles, and workplace settings.

Additional sampling produced smaller gains. Phi increased to 0.84 with 40 observations and 0.87 with 60 observations. The median observed portfolio scenario—approximately 87 observations across 9 faculty raters, 11 EPA titles, and 5 workplace settings—yielded a Phi of 0.87.

**Table 3.** Decision study projections for alternative assessment designs^a^.

Portfolio scenario	Observations	Faculty raters	EPA^b^ titles	Workplace settings	G coefficient	Phi coefficient	Absolute SEM^c^
10-observation well-distributed portfolio	10	3	3	2	0.77	0.65	0.42
20-observation well-distributed portfolio	20	5	5	3	0.86	0.76	0.32
30-observation well-distributed portfolio	30	6	6	4	0.89	0.80	0.28
40-observation well-distributed portfolio	40	8	8	4	0.91	0.84	0.25
60-observation well-distributed portfolio	60	9	10	5	0.93	0.87	0.22
Median-based observed well-distributed portfolio	87	9	11	5	0.94	0.87	0.21
100-observation well-distributed portfolio	100	10	11	5	0.94	0.88	0.21

a Each scenario represents a study-defined, well-distributed portfolio in which observations, faculty raters, EPA titles, and workplace settings vary together.

b EPA: entrustable professional activity.

c SEM: standard error of measurement.

### Faculty-Rater Breadth and Observation Density

The D-study heatmap examined the joint contribution of observation count and faculty-rater breadth while holding EPA title breadth at 6 and workplace-setting breadth at 4 (Figure 1).

Across the displayed grid, projected Phi coefficients ranged from 0.62 to 0.86. Phi coefficients increased with both larger observation counts and broader faculty-rater representation.

Under this fixed EPA and workplace-setting structure, the lowest displayed observation count reaching Phi ≥0.80 was 20 observations with 8 or 10 faculty raters. With faculty-rater breadth fixed at 6 raters, the first displayed scenario reaching Phi ≥0.80 was 30 observations. The complementary D-study curve (Figure S1 in Multimedia Appendix 1) showed that the Phi coefficient increased from approximately 0.65 at 10 observations to 0.80 at 30 observations, while the G coefficient increased from approximately 0.77 to 0.89. From 40 to 100 observations, the Phi coefficient increased from approximately 0.84 to 0.88, and the G coefficient increased from approximately 0.91 to 0.94.

**Figure 1.**
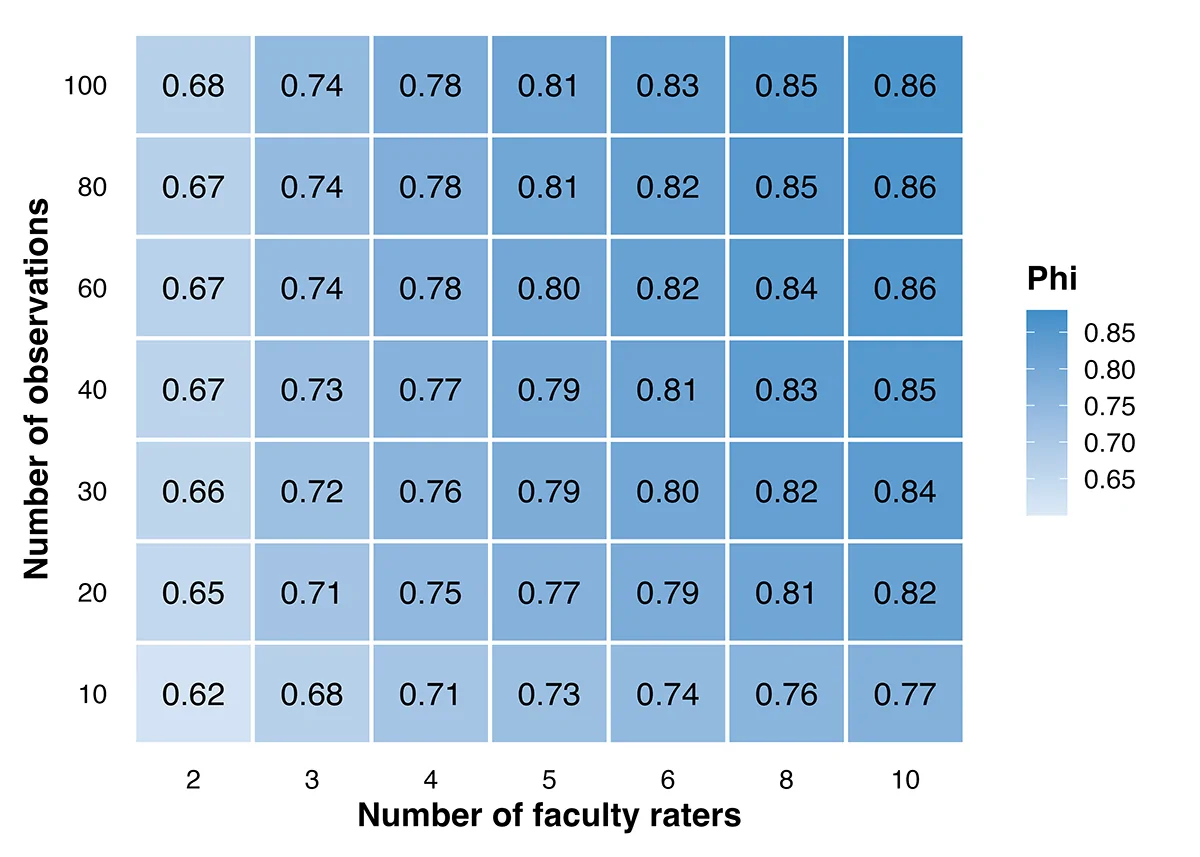
Decision-study projections of Phi coefficient by number of observations and faculty raters. Entrustable professional activity-title breadth was fixed at 6 and workplace-setting breadth was fixed at 4.

### Observed Portfolio Configurations and Concentration

Observed resident portfolios were compared with projected D-study portfolio configurations (Table 4). Most residents met the observation-count reference values, with 447 (95.9%) residents having at least 20 observations and 412 (88.4%) residents having at least 30 observations. When faculty-rater, EPA-title, and workplace-setting breadth were also considered, 398 (85.4%) residents met the 20-observation well-distributed portfolio criteria, defined as at least 20 observations, 5 faculty raters, 5 EPA titles, and 3 workplace settings. A total of 330 (70.8%) residents met the stricter 30-observation well-distributed portfolio criteria, defined as at least 30 observations, 6 faculty raters, 6 EPA titles, and 4 workplace settings. Fewer residents met the study-defined well-distributed portfolio configurations than met the corresponding raw observation-count reference values.

Resident portfolios showed some concentration. The median proportion of observations contributed by the most frequent faculty rater within each resident’s portfolio was 27.0% (IQR 21.6%‐36.7%), by the most frequent EPA title was 20.2% (IQR 15.7%‐25.0%), and by the most frequent workplace setting was 51.2% (IQR 41.4%‐64.2%).

**Table 4.** Observed resident portfolio relative to projected decision-study portfolio configurations (N=466).

Portfolio configuration	Residents, n (%)
At least 20 observations	447 (95.9)
At least 30 observations	412 (88.4)
At least 5 faculty raters	416 (89.3)
At least 6 faculty raters	377 (80.9)
At least 5 EPA^a^ titles	464 (99.6)
At least 6 EPA titles	462 (99.1)
At least 3 workplace settings	448 (96.1)
At least 4 workplace settings	402 (86.3)
20-observation well-distributed portfolio^b^	398 (85.4)
30-observation well-distributed portfolio^c^	330 (70.8)

a EPA: entrustable professional activity.

b The 20-observation well-distributed portfolio was defined as ≥20 observations, ≥5 faculty raters, ≥5 EPA titles, and ≥3 workplace settings.

c The 30-observation well-distributed portfolio was defined as ≥30 observations, ≥6 faculty raters, ≥6 EPA titles, and ≥4 workplace settings.

### Sensitivity Analyses

Sensitivity analysis results are presented in Table S1 (Multimedia Appendix 1). In the EPA title × workplace setting sensitivity model, the EPA title × workplace setting interaction accounted for 0.3% of total variance, and residual/encounter-level variance accounted for 40.2%. In the hospital-aware model, resident hospital and faculty hospital accounted for 1.1% and 0.8% of total variance, respectively. The main variance-component pattern remained stable, with resident variance accounting for 25.8%, and residual/encounter-level variance accounting for 39.8%.

In the complexity-adjusted sensitivity model, which included 45,513 observations after excluding 13 records with missing case-complexity data, advanced/nonroutine cases were associated with entrustment-supervision ratings 0.34 points lower than basic/routine cases. Residual/encounter-level variance decreased modestly from 40.1% in the primary model to 39.1% after adjusting for case complexity, while the main variance-component pattern remained stable.

## Discussion

### Principal Findings and Implications

This nationwide digital platform study found that EPA-based progression review depends on both observation density and portfolio breadth. Single EPA observations were insufficient for dependable resident-level interpretation, whereas well-distributed portfolios showed higher projected dependability. A 20-observation portfolio across 5 faculty raters, 5 EPA titles, and 3 workplace settings reached the prespecified Phi ≥0.70 psychometric reference point, while a 30-observation portfolio across 6 faculty raters, 6 EPA titles, and 4 workplace settings reached the stricter Phi ≥0.80 reference point. These findings provide internal structure validity evidence for EPA-based workplace assessment and indicate that defensible progression judgments depend not only on assessment volume but also on purposeful sampling across assessors, tasks, and contexts.

The main contribution of this study is to reframe EPA portfolio dependability as a multidimensional sampling issue in programmatic assessment [9,25]. In many CBME implementations, portfolio completeness is often discussed primarily in terms of the number of completed assessments [2]. Our findings suggest that assessment count alone is insufficient for dependable resident-level interpretation. Instead, dependability depends on how assessment evidence is distributed across faculty raters, EPA titles, and workplace settings [23,24]. This perspective is relevant to medical education, providing a practical way for programs, competency committees, and digital assessment platforms to monitor not only whether residents have enough assessment records but also whether those records are sufficiently broad for the intended progression decision [20,35,36]. Therefore, the findings support portfolio-design thinking rather than simple numeric assessment targets.

Our findings extend prior work on entrustment-derived workplace assessment. Kelleher et al [23] identified rater variance as a major source of score variation in internal medicine assessments and showed that reliability improved with aggregation across raters and encounters. Ryan et al [14] multi-institutional Core EPA study found limited learner-attributable variance in many workplace-based assessment datasets and cautioned against using such assessments alone for high-stakes summative decisions. Tanaka et al [37] similarly showed that anesthesiology EPAs differed in their capacity to discriminate resident performance. Against this background, our findings suggest that EPA-based assessment can support dependable resident-level judgments when portfolios are deliberately sampled across raters, EPAs, and workplaces. The relatively higher dependability estimates observed in this study may reflect the postgraduate specialty context, alignment between EPAs and routine clinical work, greater resident-level performance variation, or features of national implementation [12].

The generalizability of these findings should be understood in 2 ways. The analytic framework may be transferable: other EPA-based or workplace-based assessment systems can use G-study and D-study methods to examine how portfolio dependability changes with assessment count, rater breadth, EPA breadth, and workplace-setting breadth [23-27]. However, the numerical estimates are context dependent. The projected portfolio configurations in this study reflect the Taiwan otorhinolaryngology EPA framework, the E-MyWay platform structure, local rating practices, workplace settings, and the variance components observed in this dataset. Therefore, these configurations should not be interpreted as universal requirements for demonstrating competence. Specialties with more or fewer EPAs, different case-mix structures, different assessment cultures, or predominantly nonprocedural or longitudinal clinical work would require context-specific modeling.

A central implication is that observation count is necessary but insufficient. Programmatic assessment often emphasizes accumulating multiple low-stakes observations, but a portfolio concentrated within a narrow set of raters, tasks, or settings may remain vulnerable to rater bias, task specificity, and contextual restriction [38]. Our results shift the question from “How many assessments are enough?” to “What configuration of evidence is dependable enough for the intended interpretation?” For CBME systems, portfolio monitoring should therefore be multidimensional, incorporating observation density, assessor diversity, EPA breadth, and workplace-setting breadth [24,39]. Digital platforms can operationalize this principle by tracking not only portfolio size but also rater diversity, EPA coverage, and workplace-setting breadth. With the resident as the object of measurement, these projected configurations refer to aggregate resident-level portfolio dependability across EPA-based assessment records, not certification-level dependability for each individual EPA. Therefore, they should not be interpreted as evidence that assessment of a subset of EPA titles is sufficient for entrustment across all core activities.

The variance-component findings reinforce this point. Resident variance accounted for a meaningful proportion of score variation, supporting the interpretation that EPA ratings captured differences in residents’ entrustment readiness. However, resident variance should not be interpreted as a pure measure of competence, as it may reflect differences in training stage, exposure opportunity, program context, and individual performance. At the same time, residual/encounter-level variance was the largest component, and faculty rater variance and resident × faculty rater interaction variance were also substantial. This pattern should not be viewed simply as measurement failure. Workplace performance is situated, relational, and contingent on patient acuity, task complexity, clinical setting, and supervisor judgment. In surgical and procedurally intensive specialties, this contextual variability may be particularly pronounced, given that competence integrates diagnostic reasoning, procedural skill, perioperative decision-making, team coordination, and risk management. EPA ratings are therefore best understood as contextually sampled indicators of readiness rather than fixed traits revealed through isolated encounters [13].

The sensitivity analyses further supported the robustness of the primary variance structure. In the hospital-aware sensitivity analysis, resident-hospital and faculty-hospital variance components were small, and the main variance pattern remained stable after adding these contextual random effects. This finding supports the interpretation that the primary results reflected aggregate resident-level portfolio dependability rather than being driven mainly by hospital-level clustering. Nevertheless, hospital codes may not fully capture all programmatic differences in curriculum, clinical exposure, assessment culture, faculty development, or rater calibration [12,38].

The complexity-adjusted sensitivity model also supported the primary interpretation. Although advanced/nonroutine cases were associated with lower entrustment-supervision ratings, adjustment for measured case complexity only modestly reduced residual/encounter-level variance and did not materially alter the primary variance-component pattern. These findings suggest that measured case complexity contributed to rating variation but did not account for most encounter-level variability, reinforcing the need to aggregate evidence across multiple observations, faculty raters, EPA titles, and workplace settings. Future assessment implementation may reduce unexplained encounter-level variation by strengthening behavioral anchors, improving rater orientation, documenting case complexity more systematically, and ensuring that resident portfolios are sampled across multiple raters, EPA titles, and workplace settings.

This study contributes to a limited evidence base on EPA-based summative assessment in postgraduate surgical specialty training. Much of the existing reliability literature has emerged from undergraduate medical education or nonsurgical postgraduate contexts [27,40,41]. Otorhinolaryngology-head and neck surgery provides an informative test case, spanning medical and surgical practice, including acute airway and bleeding emergencies, head and neck infections, otologic and sinonasal disease, sleep-disordered breathing, head and neck tumors, and facial plastic and reconstructive conditions across emergency, inpatient, outpatient, procedural, and operating-room environments. The finding that dependable decisions are achievable only through broadly sampled portfolios suggests that EPA-based summative assessment can be psychometrically defensible in surgical specialty training when systems are designed to capture the breadth of clinical work rather than rely on convenience-based observations [42].

These findings have practical implications for clinical competency committees (CCCs). CCCs are often asked to make progression decisions from heterogeneous evidence but may lack explicit guidance for judging whether the quantitative observation evidence base is sufficiently broad. The projected portfolio configurations in this study provide model-based psychometric reference points: approximately 20 well-distributed observations may support lower-stakes resident-level portfolio review, whereas more consequential progression deliberation may require broader evidence, such as 30 observations across multiple faculty raters, EPA titles, and workplace settings. These configurations should not be applied mechanically as competence standards. CCCs must still incorporate narrative feedback, case complexity, performance trajectory, professionalism concerns, and contextual knowledge. Rather, the configurations may help committees and programs judge whether quantitative entrustment data are sufficiently distributed to support defensible deliberation [35,36].

Importantly, the projected portfolio configurations should be interpreted as model-based psychometric evidence for portfolio design, not as validated standards for competence, certification, or promotion. This cross-sectional G-study/D-study estimated internal structure evidence and projected dependability under selected sampling configurations, but it did not evaluate whether these configurations improve the accuracy, fairness, or consequences of actual progression decisions [26,34,43]. Future studies should link EPA portfolio dependability to independent educational outcomes, including CCC decisions, remediation, delayed progression, graduation outcomes, and subsequent clinical performance [35,36].

The results also speak to assessment burden [2,11]. Excessive assessment expectations may overwhelm faculty and residents, encourage superficial completion, and weaken feedback quality, whereas insufficient sampling may lead to unstable high-stakes decisions. In this dataset, the median observed resident portfolio exceeded the stricter projected portfolio configuration, suggesting feasibility within a mature national digital assessment system. Yet approximately 71% (330/466) of residents met all criteria for the stricter 30-observation scenario, despite many having sufficient raw observation counts. This gap suggests that portfolio breadth, rather than assessment volume alone, may have constrained portfolio completeness for a substantial subgroup. Thus, implementation should focus not only on increasing the number of assessments but also on monitoring portfolio distribution. A platform dashboard could flag residents who meet raw observation counts but lack sufficient rater breadth, EPA-title breadth, or workplace-setting breadth before CCC review.

Finally, this study should be interpreted as one component of a broader validity argument [34,43]. Generalizability theory provided internal structure evidence by estimating the extent to which entrustment ratings reflected resident differences relative to rater, task, context, interaction, and residual/encounter-level variation. D-studies linked this evidence to intended use by projecting resident-level dependability under alternative portfolio designs. This approach aligns with contemporary validity thinking: validity resides not in the assessment tool alone but in the interpretation and use of assessment data for a specific decision. Formative EPA observations are context-dependent assessments intended primarily to support feedback and learning within specific clinical encounters. They should not be used individually, or in small numbers, to classify a resident as performing above or below stage-specific expectations. Such classification requires longitudinal synthesis of evidence through a summative process, such as CCC deliberation. Reliability is necessary but not sufficient; content alignment, response processes, relations to other variables, consequences, and CCC deliberation remain essential [23].

### Limitations

This study has limitations. First, the numerical D-study projections were derived from one mature national digital workplace assessment platform in Taiwan otorhinolaryngology-head and neck surgery residency training. Therefore, they should not be generalized as universal portfolio requirements. The estimates are conditional on the Taiwan specialty EPA framework, E-MyWay platform structure, local rating practices, workplace settings, hospital/program context, and observed variance components. Although the hospital-aware sensitivity model showed small resident-hospital and faculty-hospital variance components, hospital codes may not fully capture program-level differences in curriculum, clinical exposure, assessment culture, faculty development, or rater calibration. Other specialties or assessment systems would require context-specific modeling.

Second, workplace-based assessment data were opportunistic and unbalanced, reflecting clinical workflow, case availability, faculty availability, resident rotation assignments, and local assessment culture rather than balanced or randomized sampling of resident performance. The portfolio concentration analysis characterized this imbalance but did not statistically correct sampling bias. Therefore, the D-study projections should be interpreted as model-based estimates from observed real-world assessment data, not as evidence from a controlled sampling design.

Third, faculty-assigned entrustment-supervision ratings were ordinal judgments but were treated as approximately numeric to permit variance decomposition and D-study projection within the classical G-theory framework. This analytic approximation simplifies the ordered categorical nature and the social complexity of entrustment judgment. Ordinal models, such as cumulative-link mixed models or many-facet ordinal models, may provide complementary evidence in future work.

Fourth, quantitative ratings do not capture the full richness of narrative feedback, resident reflection, performance trajectory, phase-of-care information, or CCC deliberation. Although case complexity was included in a sensitivity analysis, it was captured only as basic/routine versus advanced/nonroutine, and phase of care was not separately modeled. EPA12 was also excluded, owing to its inconsistent implementation across the analytic period. In addition, the D-study projections assumed well-distributed portfolios and should not be interpreted to mean that any 20 or 30 observations would yield equivalent dependability, nor that the projected configurations establish sufficient evidence for entrustment in each individual EPA.

Finally, the present study evaluated portfolio dependability and internal-structure evidence rather than whether shorter formative EPA portfolios could identify residents performing above or below stage-specific expectations. The analyzed EPA records were ad hoc, low-stakes, formative, and context dependent; each observation was intended to support feedback within a specific clinical encounter rather than summative classification of overall resident performance. Stage-relative classification generally requires longitudinal synthesis through CCC summative entrustment decisions. Although CCC decisions became available in the E-MyWay database beginning in January 2025, they were not available across the full analytic period and were not consistently temporally aligned with the formative EPA portfolios analyzed in this study. In addition, as CCC deliberations may incorporate longitudinal EPA evidence, using contemporaneous CCC decisions to define performance subgroups would require careful temporal separation and complementary outcomes to minimize circular interpretation.

### Future Directions

Future studies should prospectively align formative EPA portfolios accumulated before each CCC review with subsequent CCC summative entrustment decisions. Additional outcomes, including remediation, delayed progression, promotion, and subsequent clinical performance, should be incorporated to provide evidence beyond the EPA ratings themselves. Such longitudinal analyses could determine whether residents performing clearly above or below stage-specific expectations can be identified with fewer observations while accounting for the context-dependent nature of formative workplace assessment. These analyses should also examine whether portfolio configurations associated with prespecified dependability targets vary by postgraduate year and by individual EPA. Additional work should examine how narrative feedback, resident reflection, and case complexity can be integrated into summative validity arguments. Common scale anchors alone do not demonstrate successful rater calibration. Prospective studies should therefore use standardized cases, video-recorded encounters, or independently duplicated ratings to evaluate interrater agreement directly and determine whether structured faculty calibration improves rating consistency. Cross-specialty comparisons are needed to determine whether medical, surgical, and hybrid specialties require different sampling architectures. Finally, future research should test whether real-time learning analytics dashboards improve portfolio completeness, reduce assessment burden, and strengthen CCC preparedness.

### Conclusions

This nationwide cross-sectional G- and D-study showed that digital EPA assessment portfolios require purposeful sampling across faculty raters, EPA titles, and workplace settings to support dependable resident-level progression review. The findings support study-defined projected portfolio configurations, including a 30-observation well-distributed portfolio that reached the stricter Phi ≥0.80 psychometric reference point. These model-based dependability estimates should complement, rather than replace, narrative evidence, case complexity, performance trajectory, and CCC deliberation.

## Supplementary material

10.2196/104628 Multimedia Appendix 1 Supplementary generalizability and decision study model specifications, dependability projections, and sensitivity variance-component analyses.

10.2196/104628 Checklist 1 STROBE checklist.

## Abbreviations

CBME: competency-based medical education
CCC: clinical competency committee
D-study: decision study
EPA: entrustable professional activity
G-study: generalizability study
SEM: standard error of measurement
STROBE: Strengthening the Reporting of Observational Studies in Epidemiology
TSO-HNS: Taiwan Society of Otorhinolaryngology-Head and Neck Surgery
