# Supplementary material for: Dependability of Entrustable Professional Activity Portfolios in Otorhinolaryngology Residency: Nationwide Generalizability and Decision Study
Source: JMIR Med Educ. 2026 Oct 5;12:e104628. doi: 10.2196/104628 (PMC13637980; doi:10.2196/104628)
Supplement: Supplementary file 1 [file mededu-v12-e104628-s001.docx]

**Multimedia Appendix 1**

I. **Supplementary Methods.** Generalizability study and Decision study model specification.

**Primary G-study model**

The primary G-study was specified as an intercept-only random-effects linear mixed-effects model. The resident was treated as the object of measurement. Faculty rater, EPA title, workplace setting, resident × faculty rater interaction, resident × EPA title interaction, faculty rater × EPA title interaction, and residual variance were estimated as random components.

The model was specified as:

$$Y=\mu+R+F+E+W+RF+RE+FE+\epsilon$$

where $Y$represents the faculty-assigned entrustment–supervision level, $\mu$is the grand mean, $R$is resident, $F$is faculty rater, $E$is EPA title, $W$is workplace setting, $RF$is the resident × faculty rater interaction, $RE$is the resident × EPA title interaction, $FE$is the faculty rater × EPA title interaction, and $\epsilon$is residual / encounter-level variance.

In R, the primary model was specified as:

entrustment_level ~ 1 +

(1 | resident_id) +

(1 | faculty_id) +

(1 | epa_label) +

(1 | workplace_setting) +

(1 | resident_id:faculty_id) +

(1 | resident_id:epa_label) +

(1 | faculty_id:epa_label)

**EPA title × workplace setting sensitivity model**

The EPA title × workplace setting sensitivity model added the EPA title × workplace setting interaction to the primary G-study model:

entrustment_level ~ 1 +
 (1 | resident_id) +
 (1 | faculty_id) +
 (1 | epa_label) +
 (1 | workplace_setting) +
 (1 | resident_id:faculty_id) +
 (1 | resident_id:epa_label) +
 (1 | faculty_id:epa_label) +
 (1 | epa_label:workplace_setting)

This model examined whether specific EPA title–workplace setting combinations contributed additional score variance beyond the primary model.

**Hospital-aware sensitivity model**

The hospital-aware sensitivity model added resident hospital and faculty hospital as contextual random effects:

entrustment_level ~ 1 +
 (1 | resident_id) +
 (1 | faculty_id) +
 (1 | epa_label) +
 (1 | workplace_setting) +
 (1 | resident_id:faculty_id) +
 (1 | resident_id:epa_label) +
 (1 | faculty_id:epa_label) +
 (1 | resident_hospital_code) +
 (1 | faculty_hospital_code)

This model examined whether accounting for hospital affiliation materially changed the variance-component structure.

**Complexity-adjusted sensitivity model**

The complexity-adjusted sensitivity model added case complexity as a fixed effect to the primary G-study model:

entrustment_level ~ case_complexity +

(1 | resident_id) +

(1 | faculty_id) +

(1 | epa_label) +

(1 | workplace_setting) +

(1 | resident_id:faculty_id) +

(1 | resident_id:epa_label) +

(1 | faculty_id:epa_label)

This model examined whether adjustment for measured case complexity materially changed the variance-component structure.

**D-study projection formulas**

D-study projections were derived from the variance components estimated in the primary G-study model. Each D-study scenario represented a study-defined, well-distributed resident assessment portfolio. Therefore, projected dependability reflected both the number of observations and their distribution across faculty raters, EPA titles, and workplace settings.

The relative error variance was calculated as:

$$\sigma_{\delta}^{2}=\frac{\sigma_{RF}^{2}}{n_{F}}+\frac{\sigma_{RE}^{2}}{n_{E}}+\frac{\sigma_{\epsilon}^{2}}{n_{obs}}$$

where $\sigma_{\delta}^{2}$is the relative error variance, $\sigma_{RF}^{2}$is the resident × faculty rater variance, $\sigma_{RE}^{2}$is the resident × EPA title variance, $\sigma_{\epsilon}^{2}$is the residual / encounter-level variance, $n_{F}$is the number of faculty raters, $n_{E}$is the number of EPA titles, and $n_{obs}$is the total number of observations.

The absolute error variance was calculated as:

$$\sigma_{\Delta}^{2}=\sigma_{\delta}^{2}+\frac{\sigma_{F}^{2}}{n_{F}}+\frac{\sigma_{E}^{2}}{n_{E}}+\frac{\sigma_{W}^{2}}{n_{W}}+\frac{\sigma_{FE}^{2}}{n_{F}n_{E}}$$

where $\sigma_{\Delta}^{2}$is the absolute error variance, $\sigma_{F}^{2}$is faculty rater variance, $\sigma_{E}^{2}$is EPA title variance, $\sigma_{W}^{2}$is workplace-setting variance, $\sigma_{FE}^{2}$is faculty rater × EPA title variance, $n_{F}$is the number of faculty raters, $n_{E}$is the number of EPA titles, and $n_{W}$is the number of workplace settings.

The generalizability coefficient was calculated as:

$$G=\frac{\sigma_{R}^{2}}{\sigma_{R}^{2}+\sigma_{\delta}^{2}}$$

where $G$is the generalizability coefficient for relative decisions, $\sigma_{R}^{2}$is resident variance, and $\sigma_{\delta}^{2}$is relative error variance.

The Phi coefficient was calculated as:

$$\Phi=\frac{\sigma_{R}^{2}}{\sigma_{R}^{2}+\sigma_{\Delta}^{2}}$$

where $\Phi$is the dependability coefficient for absolute decisions, $\sigma_{R}^{2}$is resident variance, and $\sigma_{\Delta}^{2}$is absolute error variance.

The absolute standard error of measurement was calculated as:

$$SEM_{absolute}=\sqrt{\sigma_{\Delta}^{2}}$$

II. **Supplementary Figure S1.** Decision study projections of dependability across practical assessment portfolios.

III. **Supplementary Table S1.** Variance components from EPA01–EPA11 sensitivity generalizability study models.

| Variance component^a^ | Variance^b^ | % of total variance^c^ |
| --- | --- | --- |
| EPA title × workplace setting sensitivity model^d^ |  |  |
| Resident | 0.32 | 27.0 |
| Faculty rater | 0.13 | 10.8 |
| EPA title | 0.05 | 3.9 |
| Workplace setting | 0.03 | 2.6 |
| Resident × faculty rater | 0.12 | 10.3 |
| Resident × EPA title | 0.02 | 2.0 |
| Faculty rater × EPA title | 0.04 | 3.0 |
| EPA title × workplace setting | 0.00 | 0.3 |
| Residual / encounter-level variance | 0.47 | 40.2 |
| Hospital-aware sensitivity model^e^ |  |  |
| Resident | 0.31 | 25.8 |
| Faculty rater | 0.12 | 10.4 |
| EPA title | 0.05 | 4.2 |
| Workplace setting | 0.03 | 2.4 |
| Resident hospital | 0.01 | 1.1 |
| Faculty hospital | 0.01 | 0.8 |
| Resident × faculty rater | 0.12 | 10.3 |
| Resident × EPA title | 0.02 | 2.0 |
| Faculty rater × EPA title | 0.04 | 3.0 |
| Residual / encounter-level variance | 0.47 | 39.8 |
| Complexity-adjusted sensitivity model^f^ |  |  |
| Resident | 0.33 | 28.3 |
| Faculty rater | 0.14 | 11.7 |
| EPA title | 0.04 | 3.3 |
| Workplace setting | 0.03 | 2.2 |
| Resident × faculty rater | 0.13 | 10.9 |
| Resident × EPA title | 0.02 | 1.9 |
| Faculty rater × EPA title | 0.03 | 2.6 |
| Residual / encounter-level variance | 0.46 | 39.1 |

Abbreviation: EPA, entrustable professional activity.

^a^ Variance components were estimated using restricted maximum likelihood from EPA01–EPA11 records.

^b^ Variance values are rounded to two decimal places.

^c^ Percentages were calculated from unrounded variance components.

^d^ The EPA title × workplace setting sensitivity model added an EPA title × workplace setting random-effect component to the primary G-study model.

^e^ The hospital-aware sensitivity model added resident hospital and faculty hospital random-intercept components to the primary G-study model.

^f^ The complexity-adjusted sensitivity model added case complexity as a fixed effect to the primary G-study model. This model included 45,513 observations after excluding 13 observations with missing case-complexity data.
